# Polysaccharides Extracted from *Dendrobium officinale* Grown in Different Environments Elicit Varying Health Benefits in *Caenorhabditis elegans*

**DOI:** 10.3390/nu15122641

**Published:** 2023-06-06

**Authors:** Nkwachukwu Oziamara Okoro, Arome Solomon Odiba, Qi Yu, Bin He, Guiyan Liao, Cheng Jin, Wenxia Fang, Bin Wang

**Affiliations:** 1 Institute of Biological Sciences and Technology, Guangxi Academy of Sciences, Nanning 530007, China; 2 Department of Pharmaceutical and Medicinal Chemistry, University of Nigeria, Nsukka 410001, Nigeria; 3 State Key Laboratory of Mycology, Institute of Microbiology, Chinese Academy of Sciences, Beijing 100101, China; 4 School of Agriculture and Engineering, Guangxi Vocational and Technical College, Nanning 530226, China

**Keywords:** aging, *Caenorhabditis elegans*, *Dendrobium officinale*, lifespan, polysaccharides

## Abstract

*Dendrobium officinale* is one of the most widely used medicinal herbs, especially in Asia. In recent times, the polysaccharide content of *D. officinale* has garnered attention due to the numerous reports of its medicinal properties, such as anticancer, antioxidant, anti-diabetic, hepatoprotective, neuroprotective, and anti-aging activities. However, few reports of its anti-aging potential are available. Due to high demand, the wild *D. officinale* is scarce; hence, alternative cultivation methods are being employed. In this study, we used the *Caenorhabditis elegans* model to investigate the anti-aging potential of polysaccharides extracted from *D. officinale* (DOP) grown in three different environments; tree (TR), greenhouse (GH), and rock (RK). Our findings showed that at 1000 µg/mL, GH-DOP optimally extended the mean lifespan by 14% and the maximum lifespan by 25% (*p* < 0.0001). TR-DOP and RK-DOP did not extend their lifespan at any of the concentrations tested. We further showed that 2000 µg/mL TR-DOP, GH-DOP, or RK-DOP all enhanced resistance to H_2_O_2_-induced stress (*p* > 0.05, *p* < 0.01, and *p* < 0.01, respectively). In contrast, only RK-DOP exhibited resistance (*p* < 0.01) to thermal stress. Overall, DOP from the three sources all increased HSP-4::GFP levels, indicating a boost in the ability of the worms to respond to ER-related stress. Similarly, DOP from all three sources decreased α-synuclein aggregation; however, only GH-DOP delayed β-amyloid-induced paralysis (*p* < 0.0001). Our findings provide useful information on the health benefits of DOP and also provide clues on the best practices for cultivating *D. officinale* for maximum medicinal applications.

## 1. Introduction

In recent years, the traditional Chinese medicinal herb *Dendrobium officinale* has gained significant interest for its pharmacological properties [1]. As a rare perennial orchid, *D. officinale* has a wide geographical spread across several countries, such as China, Australia, India, the United States of America, and Japan [2,3]. This herb has been shown to have a variety of health benefits, including anticancer [4], antiangiogenic [5], anti-inflammatory [6], antioxidant [7], anti-diabetic [8,9], immunity-enhancing [10], hepatoprotective [11,12], and neuroprotective activities [2]. Studies have indicated that the polysaccharides found in *D. officinale* are some of the bioactive chemical constituents responsible for its medicinal benefits, including antioxidant, blood lipid and sugar lowering, hepatoprotective, and anti-aging activities [13,14,15,16,17,18]. Additionally, the polysaccharides have been demonstrated to have anticancer effects on gastric cancer cells and protective effects on experimental gastric ulcers in mice [13,19]. Furthermore, *D. officinale* polysaccharides (DOP) therapy attenuated hepatic lipid metabolic disorders and relieved symptoms of hepatic lipid accumulation in type 2 diabetes in rats [11,16].

The wild *D. officinale* is highly endangered because it requires a specific growth profile and a long time to mature [20,21,22]. Moreover, due to the high market demand, the wild *D. officinale* has been excessively exploited, resulting in its scarcity. Therefore, attempts have been made to cultivate *D. officinale* in controlled environments such as greenhouses to meet market demands and conserve natural supplies [20,21,22]. Studies have reported that bioactive constituents such as polysaccharides, phenolic acids, and flavones are the primary components responsible for the health benefits of *D. officinale*, and their concentration fluctuates when the growth environment (such as nutrients, temperature, and light availability) is altered [20,21,22].

As *D. officinale* has anti-aging potential, it is necessary to explore its active compounds with anti-aging effects. The free-living worm *Caenorhabditis elegans* is a well-established model for aging research because it shares conserved pathways and similar hallmarks of aging with humans, and about two-thirds of the human genome is well-conserved in the worm [23,24,25,26]. In this study, we employed the *C. elegans* model to investigate the potential health benefits of *D. officinale* polysaccharides (DOP) extracted from *D. officinale* grown in three different environments: on trees, in a greenhouse, and on rocks. Our findings revealed that DOP from *D. officinale* cultivated in different environments exerted varying effects on the health of *C. elegans*. Our results further suggest that *D. officinale* metabolism differs across these environments, which may have implications for the medicinal use of this plant. Our findings may help inform the cultivation of *D. officinale* for optimal therapeutic benefits.

## 2. Materials and Methods

### 2.1. Plant Material

*D. officinale* fresh stems from three cultivation environments, including trees, greenhouses and rocks, were obtained from Yandang Mountain, Wenzhou, Zhejiang Province, China. The *D. officinale* stems were carefully cleaned, washed with distilled water, and dried in the 80 °C incubator for 24 h. The dried stems were then ground into powder and stored in a −20 °C freezer.

### 2.2. C. elegans Strains and Maintenance

*C. elegans* strains used in this study include: N2-Bristol (wild type), SJ4005(*zcIs4 [hsp-4::GFP]V*), CL4176*(smg-1(cc546)I;dvIs27X)*, and NL5901*(pkIs2386[unc54p::alphasynuclein::YFP+ unc-119(+)])*. *Escherichia coli* (OP50) was obtained from the Caenorhabditis Genetics Centre (CGC) in Minneapolis, MN, USA as a food source. The strains were cultured, maintained, and assayed at 20 °C (unless otherwise stated) on nematode growth medium (NGM) plates seeded with *E. coli* (OP50) bacteria according to established methods [27]. All worm strains used in the assays were cultured in the presence of sufficient food to avoid starvation for at least 3 generations before use. Synchronized populations of worms for all experiments were obtained by filtering a mixed population of properly maintained worms through an 11 μm pore-sized membrane filter (Merck Millipore Ltd., Lowe, NJ, USA) using M9 buffer to obtain the L1 larvae, which were subsequently cultured to L4 stage before use in assays that require L4 stage.

### 2.3. Preparation and Purification of DOP

To prepare *D. officinale* polysaccharides (DOP), 500 mL of water was added to 0.5 g *D. officinale* stem powder and extracted twice at 75 °C for 4 h [28,29]. The resulting mixture was filtered to collect the supernatant, which was then concentrated to 50 mL. To obtain a final volume fraction of 75% (*v*/*v*) ethanol in the solution, the concentrated supernatant was mixed with ethanol at a ratio of 1:3. The mixture was then refrigerated at 4 °C for 8 h, followed by centrifugation at 3500 rpm for 6 min to separate the residue from the supernatant. Finally, the pellet was freeze-dried under a vacuum to yield DOP.

### 2.4. Lifespan Assay

Synchronized L4 populations of N2 worms were transferred to NGM plates containing OP50 with varying concentrations of DOP. Worms were transferred to fresh preparations every 2 days to prevent the mixing of progeny produced by the tested worms during the reproductive period. Afterward, the worms were transferred every 3 days to ensure the presence of fresh food. From the first day of adulthood both alive and dead worms were counted, and the dead worms were picked out daily. A worm was considered dead if it failed to respond to gentle stimulation with a worm pick applied multiple times [30]. Censorship was applied to worms that emerged from the petri dish and dried out on the side. Each treatment was replicated three times with 50 worms per plate, and three independent trials.

### 2.5. Hydrogen Peroxide-Induced Stress Tolerance Assay

Age-synchronized L4 worms were grown in the absence or presence of 2000 µg/mL DOP from the three sources. On the fifth day of adulthood, the worms were transferred to new NGM plates containing 5 mM hydrogen peroxide. After incubation at 20 °C for 1 h, the viability of the worms was scored until all worms were dead [31]. The assay was performed in triplicate with 30 worms per plate, and three independent repeats were carried out.

### 2.6. Thermal Stress Tolerance Assay

Age-synchronized L4 worms were cultured in the absence or presence of 2000 µg/mL DOP from the three sources. On the fifth day of adulthood, the worms were transferred to NGM plates and subjected to a temperature of 35 °C [32]. The status of the worms (alive, dead, or censored) was recorded until the end of the experiment. The assay was conducted in triplicate with 50 worms per plate, and three independent trials.

### 2.7. Endoplasmic Reticulum Stress Response Assay

Tunicamycin is a chemical that inhibits N-linked glycosylation, leading to an accumulation of misfolded proteins in the endoplasmic reticulum (ER) [33]. To induce stress in *C. elegans*, synchronized L1 larvae of the SJ4005 *(hsp-4::gfp)* strain were grown in various concentrations of DOP from the three sources and the control. On days 1, 3, and 4 of adulthood, 50 worms were placed in a 25 ng/µL tunicamycin M9 buffer and incubated at 20 °C for 4 h [34]. The worms were then immobilized with 5 µM levamisole on a 2% agarose pad on a glass slide, covered with a coverslip, and imaged using a DM6B fluorescence microscope with the GFP filter (Leica, Wetzlar, Germany). The fluorescence intensity was quantified using ImageJ software [35]. The assay was performed in triplicate for three independent trials.

### 2.8. Analysis of α-Synuclein Protein Aggregation

The aggregation of α-synuclein::YFP was examined in the NL5901 *(pkIs2386)* strain cultured with 2000 µg/mL DOP from the three sources. After 2 days culture from the L4 stage, forty adult worms were immobilized with 5 µM levamisole on 2% agarose pads and α-synuclein::YFP aggregation images were captured [36] using a Leica DM6B fluorescence microscope. The fluorescence intensity of α-synuclein::YFP was quantified using ImageJ software [35]. This assay was performed in three independent trials.

### 2.9. Amyloid-β-Induced Paralysis Assay

The effects of DOPs from three different sources on amyloid-β-induced paralysis were investigated in the CL4176 transgenic strain, which expresses the human amyloid-β protein. Synchronized L1 populations were cultured in the presence of 2000 µg/mL DOP and controls at 15 °C until the L3 larvae stage. The worms were then shifted to 25 °C to induce Aβ(1-42) expression. The number of paralyzed worms was counted at 2 h intervals until all of the worms were paralyzed [37]. The assay was performed in three independent trials.

### 2.10. Statistical Analysis

The experimental data were analyzed using GraphPad Prism 8.0 software, and the results were presented as mean ± standard error of mean (SEM) for three independent trials. To determine the statistical significance of the differences between groups in lifespan, oxidative and thermal stress resistance, and Aβ-induced toxicity assays, the log-rank test of the Kaplan–Meier survival analysis was used. For other data sets, one-way ANOVA was performed to determine statistical significance. The threshold for statistical significance was set at *p* < 0.05.

## 3. Results

### 3.1. DOP from D. officinale Cultivated in Greenhouse Extends the Lifespan of C. elegans

**Figure 1:**
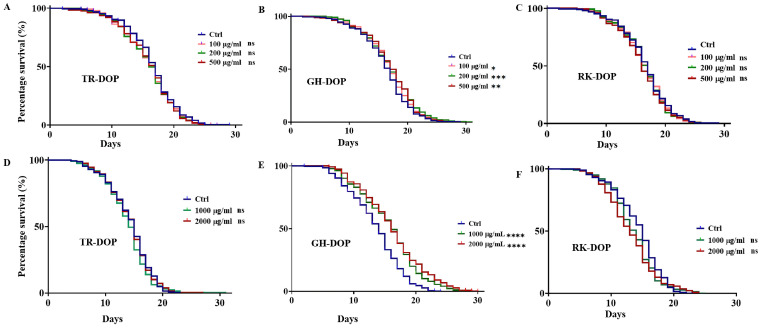
Lifespan modulating effect of DOP from *D. officinale* cultivated in different environments. (**A**–**C**) Survival curves of N2 worms treated with 100 µg/mL, 200 µg/mL and 500 µg/mL of TR-DOP, GH-DOP and RK-DOP, respectively, show a significant increase in the mean lifespan for GH-DOP compared to the control (Table S1). No increase in mean lifespan was observed for TR-DOP and RK-DOP. (**D**–**F**) Survival curves of N2 worms treated with 1000 µg/mL and 2000 µg/mL of TR-DOP, GH-DOP, and RK-DOP, respectively, show a significant increase in lifespan only for GH-DOP compared to the control (Table S1). No increase in mean lifespan was observed for TR-DOP or RK-DOP. Statistical significance of one-way ANOVA is defined as, **** *p* < 0.0001, *** *p* < 0.001, ** *p* < 0.01, * *p* < 0.05 and ns represents *p* > 0.05. Three independent trials were performed with 150 worms per condition.

Previous studies on the effect of DOP in *C. elegans* established a range of effective concentrations [38,39], from which we selected 100, 200 and 500 µg/mL (Figure 1, Table S1). Our results showed that only GH-DOP significantly extended the lifespan of the worms by 5.9% (*p* < 0.01) at 200 µg/mL, and 5.9% (*p* < 0.001) at 500 µg/mL compared to the control group (Figure 1A–C). We increased the concentration of the DOPs to 1000 and 2000 µg/mL and tested the lifespan extension. TR-DOP and RK-DOP did not extend the lifespan of the worms (Figure 1D,F, Table S1). Interestingly, the lifespan-extending effect of GH-DOP was further improved to 14% at both 1000 and 2000 µg/mL concentrations, with maximum lifespan increase to 25% and 21%, respectively, compared to the control (Figure 1E, Table S1). Overall, these results suggest that GH-DOP has the potential to extend the lifespan of N2 worms. 

### 3.2. DOP Enhanced Resistance to Oxidative and Thermal Stress in C. elegans

**Figure 2:**
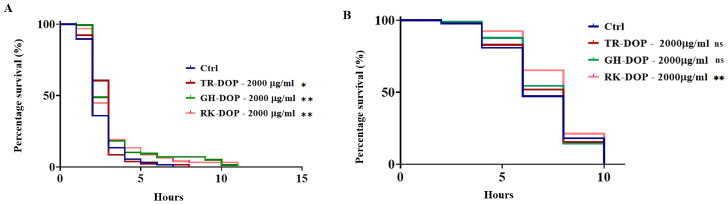
DOP enhanced stress tolerance in *C. elegans*. (**A**) Survival curve of N2 worms treated with 2000 µg/mL TR-DOP, GH-DOP and RK-DOP, and exposed to 5 mM H_2_O_2_ shows a significant resistance to oxidative stress (* *p* < 0.05, ** *p* < 0.01 and ** *p* < 0.01, respectively). Sample sizes (n) = 131, 128, 122, and 127 for the Ctrl, TR-DOP, GH-DOP and RK-DOP, respectively. (**B**) RK-DOP promotes resistance to thermal stresses (** *p* < 0.01) compared to the control, while no enhancement was observed for TR-DOP and GH-DOP (*p* > 0.05). Sample sizes (n) = 182, 187, 180, and 170 for the Ctrl, TR-DOP, GH-DOP and RK-DOP, respectively. The result represents the cumulative of three biological trials.

Given that longevity and stress tolerance are mechanistically and phenotypically linked, we further investigated the effect of DOP treatment on stress tolerance in N2 worms. Our results demonstrated that TR-DOP, GH-DOP, and RK-DOP at a dose of 2000 µg/mL significantly enhanced resistance to H_2_O_2_ when compared to the control (*p* < 0.05, *p* < 0.01 and *p* < 0.01, respectively) (Figure 2A, Table S2). In addition, we evaluated the ability of N2 worms pretreated with 2000 µg/mL DOP to tolerate thermal stress by incubating them at 35 °C. Interestingly, only RK-DOP treatment resulted in a significant increase in thermal stress resistance (*p* < 0.01), compared to the control group (Figure 2B, Table S3).

### 3.3. DOP Upregulates ER Unfolded Protein Response in C. elegans

**Figure 3:**
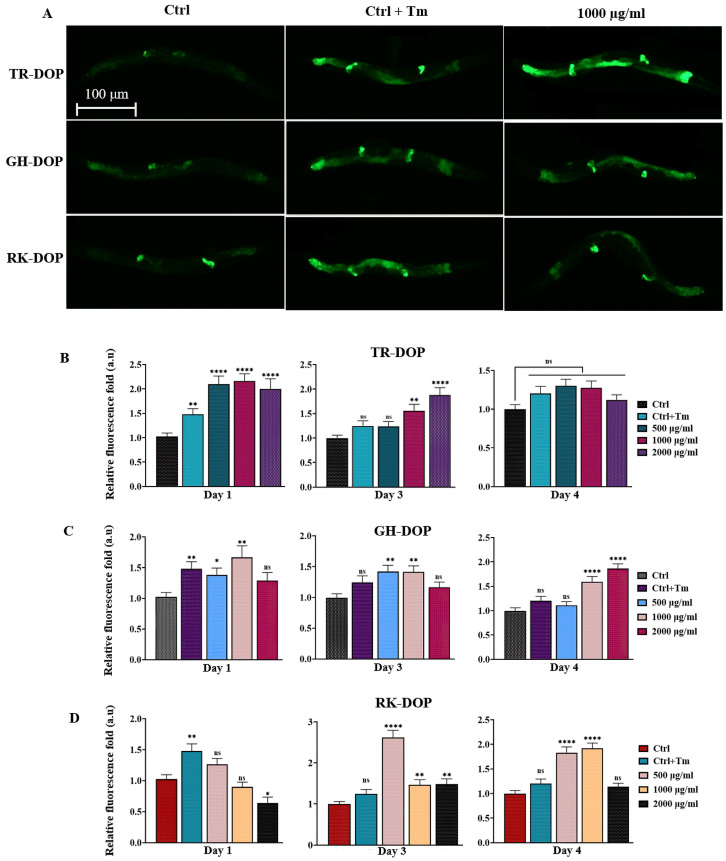
DOP upregulates ER unfolded protein response. (**A**) Representative images of day 1 adults of SJ4005 (*hsp-4::gfp*) transgenic worms treated with or without tunicamycin (Tm), and with 1000 µg/mL DOP from the three sources. (**B**–**D**) Quantification of HSP-4::GFP levels shows that DOP modulated the amount of HSP-4. Data were compared with the Tm-untreated control and presented as a fold ratio (Table S4). Images were captured using a Leica DM6B fluorescence microscope using a 5× objective (scale bar = 100 µm), and fluorescence intensity was quantified using ImageJ software. Data were analyzed using one-way ANOVA (**** *p* < 0.0001, ** *p* < 0.01, * *p* < 0.05, ns represents *p* > 0.05). The result represents the cumulative of three biological trials.

The ability of DOP to enhance ER stress response was investigated in the SJ4005 transgenic strain expressing HSP-4::GFP. Results showed that all three sources of DOP increased HSP-4::GFP levels, indicating a boost in the worms’ ability to respond to ER-related stress. However, the activity varied depending on the DOP source, concentration, and day of adulthood. The TR-DOP group showed the best response on day 1 of adulthood (Figure 3B, Table S4), while the GH-DOP and RK-DOP groups exhibited a more obvious response as the worms aged (day 4) (Figure 3C,D). Interestingly, although the ability to mount an ER unfolded protein response declined with age and was not significant (*p* > 0.05) on day 4 of adulthood in the control group, TR-DOP, GH-DOP (at 1000 and 2000 µg/mL) and RK-DOP (at 500 and 1000 µg/mL) elicited an ER unfolded protein response on day 4 of adulthood (*p* < 0.0001).

### 3.4. DOP Reduced α-Synuclein Aggregation and Delayed Amyloid-β-Induced Paralysis in C. elegans

**Figure 4:**
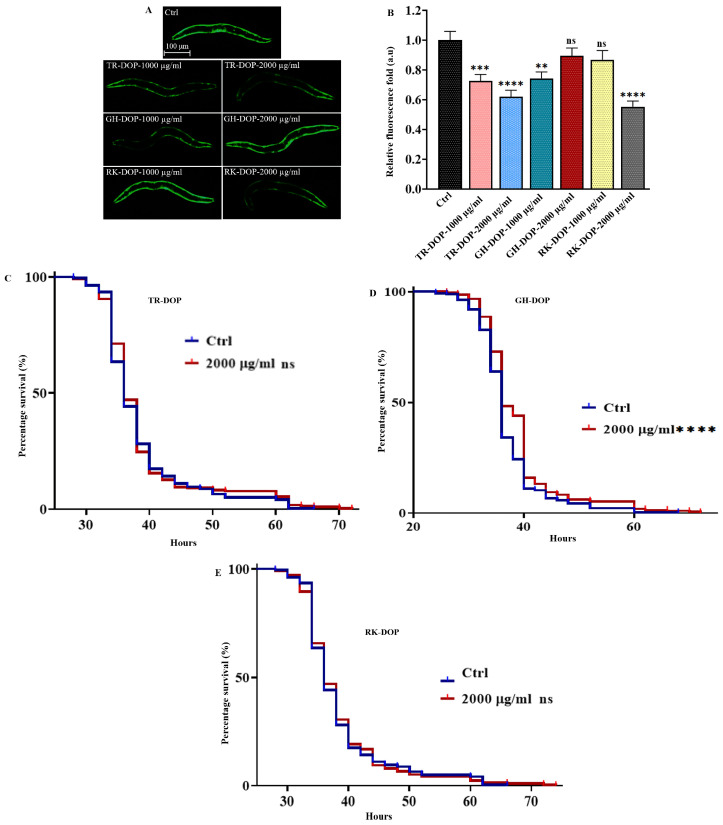
DOP reduced α-synuclein aggregation and delayed amyloid-β induced paralysis in *C. elegans*. (**A**) Representative images of human α-synuclein fused to the yellow fluorescent protein (YFP) in NL5901 transgenic worms that were treated with 1000 and 2000 µg/mL DOP. DOP from different sources exerted varying α-synuclein aggregation-reducing effects. (**B**) Fluorescence intensity quantification shows that, with the exception of GH-DOP at 2000 µg/mL and RK-DOP at 1000 µg/mL, DOP from various sources significantly reduced α-synuclein aggregation in the *NL5901* transgenic strain compared to the control. Images were captured using a Leica DM6B fluorescence microscope using a 5× objective (scale bar = 100 µm), and fluorescence intensity was quantified using ImageJ software. The data were analyzed using one-way ANOVA. Sample size n = 135, 137, 131, 122, 125, 123, and 132, respectively. (**C**–**E**) Survival curve of *CL4176* transgenic worms treated with 2000 µg/mL TR-DOP, GH-DOP, and RK-DOP (Table S5). All results represent the cumulative of three trials. (**** *p* < 0.0001, *** *p* < 0.001, ** *p* < 0.01, ns represents *p* > 0.05).

Previous studies have suggested that compounds possessing antioxidative and lifespan extension capabilities can be effective in treating neurodegenerative diseases such as Parkinson’s disease [40,41,42]. For instance, *Dendrobium nobile* alkaloid (DNLA) has demonstrated the ability to reduce neuronal damage in cultured rat primary neurons subjected to oxygen-glucose deprivation/reperfusion (OGD/RP) in vitro [43,44]. In this study, we investigated the anti-Parkinson’s effect of DOP using the *C. elegans* transgenic strain *NL5901*, which expresses human α-synuclein fused to the yellow fluorescent protein (YFP) in body-wall muscle cells [45,46,47]. We treated the NL5901 strain with 1000 and 2000 µg/mL of TR-DOP, GH-DOP, and RK-DOP, and quantified the fluorescence intensity on day 2 of adulthood (48 h post L4). The results showed that TR-DOP at both concentrations, GH-DOP at 1000 µg/mL and RK-DOP at 2000 µg/mL all significantly reduced *α*-synuclein aggregation (Figure 4A,B).

Additionally, oxidative stress has been identified as a key factor in the onset and progression of other neurodegenerative illnesses, such as Alzheimer’s disease (AD) [48,49,50]. Amyloid-beta peptide accumulation in the brain has been associated with Alzheimer’s disease and may be an early toxic event in dementia, paralysis, and stroke [51,52,53]. We investigated the effect of DOP from the three different sources on amyloid-induced paralysis in the CL4176 *C. elegans* transgenic strain. Our findings revealed that only GH-DOP significantly delayed amyloid-induced paralysis (*p* < 0.0001), whereas TR-DOP and RK-DOP did not (*p* > 0.05) (Figure 4C–E, Table S5). These results suggest the potential anti-neurodegenerative effects of DOP.

## 4. Discussion

*Dendrobium officinale* has been reported to possess several pharmacological properties notably, anticancer [3,4], hepatoprotective [4,11,12], anti-inflammatory [6], antioxidant [7,38,39], anti-diabetic [2], antiangiogenic [2,3,4,54], immuno-enhancing [3,10], and neuroprotective activities [2,44,55]. Many studies have indicated that the bioactive constituents in *D. officinale*, such as its phenolic acids, flavones and polysaccharides are the main components responsible for the health benefits [7,21,22,55,56]. Remarkably, the polysaccharides found in *D. officinale* (DOP) have been reported to elicit blood lipid-lowering, sugar-lowering, antioxidant, hepatoprotective, and anti-aging activities [13,14,15,16,17,18]. Particularly, they have been shown in mice to have anticancer effects on gastric cancer cells [13,19]. Furthermore, DOP therapy decreased hepatic lipid metabolic disorders and abated symptoms of hepatic lipid accumulation in type 2 diabetes in rats [11,16]. Despite the very promising potential of DOP against many diseases, studies on its potential for lifespan extension and anti-neurodegeneration are lacking. However, few studies have reported on the anti-aging role of DOP, and there is still much to be explored regarding its effects on the aging processes [16,17]. In this study, we used the *C. elegans* model to investigate the anti-aging potential of DOP extracted from three different growth environments: tree, greenhouse, and rock. The average amount of polysaccharides extracted varied between the various sources, with trees having the highest and rock the lowest (Table 1). We tested the lifespan-extending effects of the DOPs in wild type *C. elegans* and found that only the DOP extracted from the greenhouse extended the lifespan of the worms at all the concentrations tested (Figure 1). Polysaccharides from the marine crop *Pyropia haitanensis* have also been reported to extend lifespan in *C. elegans* by inhibiting protein aggregation [57]. Similarly, Polysaccharide from *Astragalus* has also been shown to extend lifespan in *Drosophila* through an antioxidant and IIS-dependent mechanism [58].

According to the oxidative stress theory, aging is caused by a gradual accumulation of oxidative damage to macromolecules, leading to a decline in physiological processes that worsens with age and is linked to life expectancy [36,37,38,39]. Many substances that increase lifespan in *C. elegans* also promote oxidative stress resistance [59,60,61,62]. Based on the lifespan-extending activity observed for GH-DOP, we further investigated the ability of DOP to improve tolerance to H_2_O_2_-induced oxidative stress in *C. elegans* at 2000 µg/mL. Our results showed that TR-DOP, GH-GOP and RK-GOP all elicited enhanced resistance to H_2_O_2_-induced oxidative stress (*p* < 0.05, *p* < 0.01 and *p* < 0.01, respectively) (Figure 3A). Our result is corroborated by a previous study, where DOP treatment was shown to rescue oxidative stress-induced damage in vitro in mice bone marrow mesenchymal stem cells (BMSCs) [17]. Similarly, DOP was shown to provide protective effects against H_2_O_2_-induced injury in rat H9c2 cardiomyocytes, indicating its anti-oxidative capability [63]. Antioxidant activity was also reported as one of the mechanisms via which polysaccharides from *Astragalus* extended the lifespan in Drosophila [57]. Additionally, we tested the heat tolerance of N2 worms treated with 2000 µg/mL DOP and found that only RK-DOP-treated worms showed significantly better resistance to thermal stress (*p* < 0.01) compared to the control group (Figure 3B). This difference could be attributed to the environment, as *D. officinale* grown on the rock (in the wild) is often devoid of shade, making it more vulnerable to harsh conditions, and therefore developing survival mechanisms. The molecular adaptations that enabled the plant to cope in such conditions may be responsible for the thermal resistance ability of the worms.

It has been shown in previous studies that ER stress response declines as the worm ages and usually begins early in adulthood [34]. The heat shock protein HSP-4 functions in the ER Unfolded Protein Response (UPR^ER^). Prolonged ER stress can cause obesity, atherosclerosis, diabetes, cancer, and neurological disorders [64,65,66,67,68]. To investigate the potential UPR^ER^-modulating property of DOP, we induced ER stress in DOP-treated UPR^ER^ reporter strain SJ4005 (*hsp-4::gfp*) using tunicamycin, and analyzed the fluorescence intensity. Our results showed that DOP from all three sources enhanced HSP-4 levels, although the extent varied with the source of DOP, concentration, and the age of the worm. While the effect of TR-DOP was more pronounced in early adulthood (day 1), the effects of GH-DOP and RK-DOP became more evident later as the worms aged (days 3 and 4). While studies on the effect of polysaccharides on HSP-4 are limited, a polysaccharide from *Astragalus*, a plant native to Asia has been shown to extend the lifespan of the Silkworm *Bombyx mori* via mitigating endoplasmic reticulum stress [69]. Taken together, our findings suggest that DOP enhances ER stress response. Although TR-DOP and RK-DOP did not extend the lifespan of the worms, both of them enhanced resistance to H_2_O_2_-induced oxidative stress and UPR^ER^.

Aging is a well-established risk factor for neurodegeneration, and there is evidence that amelioration of neurodegeneration has been accompanied by lifespan extension, and vice versa [70]. Loss of cellular proteostasis during aging leads to the accumulation of misfolded proteins and proteotoxicity; a hallmark of neurodegenerative disorders. Parkinson’s disease is characterized by α-synuclein aggregation and dopaminergic neuron loss in the brain, while Alzheimer’s disease is characterized by β-amyloid peptide deposition and tau neurofibrillary protein tangles [51,52,53]. Compounds such as tambulin and epigallocatechin gallate (EGCG), which have been shown to increase lifespan and exhibit anti-oxidative activity, have also been effective against neurodegenerative diseases such as Parkinson’s disease [70]. Although only a few studies have investigated the neuroprotective benefits of *Dendrobium* species, bibenzyl derivatives, alkaloids, and phenolic glucosides have been implicated [71]. There is enormous evidence that shows that polysaccharides from many sources including DOPs elicit various anti-neurodegenerative effects [55,56,72,73]. In this study, we tested the effects of the DOPs on two common markers of neurodegenerative diseases, Parkinson’s and Alzheimer’s diseases. We first investigated the anti-Parkinson’s effect of DOP in the *C. elegans* transgenic strain NL5901 and showed that DOP reduced α-synuclein aggregation in the worms, except for GH-DOP at 2000 µg/mL and RK-DOP at 1000 µg/mL (Figure 4A,B). Furthermore, the amyloid-induced paralysis in CL4176 transgenic strain was used to investigate the anti-Alzheimer’s effect of DOP. Interestingly, we found that GH-DOP significantly (*p* < 0.0001) delayed amyloid-induced paralysis while TR-DOP and RK-DOP did not (*p* > 0.05) (Figure 4C–E). These results are consistent with the lifespan extension effect of DOP (Figure 1A–F), suggesting that GH-DOP may be effective in extending lifespan and protecting against neurodegeneration. However, further studies are needed to understand the underlying mechanisms of GH-DOP’s effects.

## 5. Conclusions

Our study aimed to investigate the health-promoting effect of DOP extracted from *D. officinale* cultivated in three different environments: tree, greenhouse, and rock. Our results showed that only GH-DOP extended the lifespan of *C. elegans*, and DOP extracted from all three sources enhanced resistance to H_2_O_2_-induced oxidative stress, consistent with the widely established link between aging and oxidation. Additionally, only the RK-DOP was demonstrated to boost the worms’ ability to resist thermal stress. We further showed the DOP ameliorated key indicators of aging-associated neurodegeneration, including Parkinson’s and Alzheimer’s diseases. Overall, our findings suggest that anti-aging benefits of DOP depend on the cultivation environment, providing a reference for optimal cultivation of *D. officinale* for optimal medicinal benefits.

## Figures and Tables

**Table 1:** Yield of DOP obtained from the three cultivation sources.

Source	DO/g ^†^	DOP/mg ^†^
TR	0.50 ± 0.00 ^a^	193.63 ± 0.40 ^a^
GH	0.50 ± 0.00 ^a^	183.57 ± 1.27 ^b^
RK	0.50 ± 0.00 ^a^	142.73 ± 2.50 ^c^

Values represent Mean ± STD, n = 3 per group. ^†^ Values (column-wise) with the same letter superscripts are not statistically different, while those with a different letter are statistically different.

## Data Availability

The data presented in this study are available in the article.

## Supplementary Materials

The following supporting information can be downloaded at: https://www.mdpi.com/article/10.3390/nu15122641/s1, Table S1: Lifespan increase elicited by DOP obtained from the three cultivation sources; Table S2: Resistance to oxidative stress elicited by DOP obtained from the three cultivation sources; Table S3: Thermal stress tolerance elicited by DOP obtained from the three cultivation sources; Table S4: Changes in HSP-4::GFP levels elicited by DOP obtained from the three cultivation sources; Table S5: Anti-paralysis effect elicited by DOP obtained from the three cultivation sources.
