# Supplementary material for: Polysaccharides Extracted from Dendrobium officinale Grown in Different Environments Elicit Varying Health Benefits in Caenorhabditis elegans
Source: Nutrients. 2023 Jun 6;15(12):2641. doi: 10.3390/nu15122641 (PMC10301227; doi:10.3390/nu15122641)
Supplement: Supplementary file 1 [file nutrients-15-02641-s001.zip › nutrients-2420180-supplementary.pdf]

Table S1: Lifespan increase elicited by DOP obtained from the three cultivation sources

Culture	n value	P value (vs Ctrl)	Percentage increase in lifespan
Ctrl	411	-	-
TR-DOP (100 μg/ml)	411	> 0.05	-
TR-DOP (200 μg/ml)	411	> 0.05	-
TR-DOP (500 μg/ml)	412	> 0.05	-

Ctrl	401	-	-
TR-DOP (1000 μg/ml)	412	> 0.05	-
TR-DOP (2000 μg/ml)	415	> 0.05	-

Ctrl	498	-	-
GH-DOP (100 μg/ml)	530	0.0172	-
GH-DOP (200 μg/ml)	506	0.0002	-
GH-DOP (500 μg/ml)	503	0.0020	6%

Ctrl	353	-	-
GH-DOP (1000 μg/ml)	416	< 0.0001	14%
GH-DOP (2000 μg/ml)	421	< 0.0001	14%

Ctrl		-	-
RK-DOP (100 μg/ml)	394	> 0.05	-
RK-DOP (200 μg/ml)	395	> 0.05	-
RK-DOP (500 μg/ml)	399	> 0.05	-

Ctrl	353	-	-
RK-DOP (1000 μg/ml)	412	> 0.05	-
RK-DOP (2000 μg/ml)	404	> 0.05	-

Table S2: Resistance to oxidative stress elicited by DOP obtained from the three cultivation sources

Culture	n value	P value (vs Ctrl)	Median survival (h)	Maximum survival (h)
Ctrl	131	**-**	**3**	**7**
TR-DOP (2000 μg/ml)	128	0.0341	**3**	**9**
GH-DOP (2000 μg/ml)	122	0.0013	**3**	**11**
RK-DOP (2000 μg/ml)	127	0.0081	**3**	**11**

Table S3: Thermal stress tolerance elicited by DOP obtained from the three cultivation sources

Culture	n value	P value (vs Ctrl)	Median survival (h)	Maximum survival (h)
Ctrl	182	**-**	**6**	**10**
TR-DOP (2000 μg/ml)	187	0.8572	**8 (33%)**	**10**
GH-DOP (2000 μg/ml)	180	0.5578	**8 (33%)**	**10**
RK-DOP (2000 μg/ml)	170	0.0077	**8 (33%)**	**10**

Table S4: Changes in HSP-4::GFP levels elicited by DOP obtained from the three cultivation sources

	Culture	n value	P value (vs Ctrl)
Day 1	Ctrl	107	-
	Ctrl + Tm	108	0.0033
	TR-DOP (500 µg/ml)	109	< 0.0001
	TR-DOP (1000 µg/ml)	115	< 0.0001
	TR-DOP (2000 µg/ml)	110	< 0.0001
Day 3	Ctrl	107	-
	Ctrl + Tm	91	0.3537
	TR-DOP (500 µg/ml)	115	0.3367
	TR-DOP (1000 µg/ml)	93	0.0024
	TR-DOP (2000 µg/ml)	100	< 0.0001
Day 4	Ctrl	81	-
	Ctrl + Tm	94	0.4646
	TR-DOP (500 µg/ml)	107	0.0836
	TR-DOP (1000 µg/ml)	107	0.1404
	TR-DOP (2000 µg/ml)	108	0.8612
Day 1	Ctrl	107	-
	Ctrl + Tm	108	0.0033
	GH-DOP (500 µg/ml)	128	0.0205
	GH-DOP (1000 µg/ml)	120	0.0070
	GH-DOP (2000 µg/ml)	122	0.2894
Day 3	Ctrl	107	-
	Ctrl + Tm	91	0.1444
	GH-DOP (500 µg/ml)	86	0.0033
	GH-DOP (1000 µg/ml)	106	0.0018
	GH-DOP (2000 µg/ml)	97	0.4558
Day 4	Ctrl	81	-
	Ctrl + Tm	94	0.3540
	GH-DOP (500 µg/ml)	104	0.8016
	GH-DOP (1000 µg/ml)	108	0.0001
	GH-DOP (2000 µg/ml)	99	0.0001
Day 1	Ctrl	107	-
	Ctrl + Tm	108	0.0028
	RK-DOP (500 µg/ml)	122	0.2105
	RK-DOP (1000 µg/ml)	122	0.7235
	RK-DOP (2000 µg/ml)	117	0.0111
Day 3	Ctrl	107	-
	Ctrl + Tm	91	0.1535
	RK-DOP (500 µg/ml)	94	<0.0001
	RK-DOP (1000 µg/ml)	106	0.0027
	RK-DOP (2000 µg/ml)	101	0.0025
	Ctrl	81	-

	Ctrl + Tm	94	0.2433
Day 4	RK-DOP (500 μg/ml)	95	<0.0001
	RK-DOP (1000 μg/ml)	104	<0.0001
	RK-DOP (2000 μg/ml)	111	0.4737

Table S5: Anti-paralysis effect elicited by DOP obtained from the three cultivation sources

Culture	n value	P value (vs Ctrl)
Ctrl	217	-
TR-DOP (2000 μg/ml)	219	0.5925
Ctrl	272	-
GH-DOP (2000 μg/ml)	362	<0.0001
Ctrl	217	-
RK-DOP (2000 μg/ml)	213	0.8837
